# A novel disulfide death-related genes prognostic signature identifies the role of IPO4 in glioma progression

**DOI:** 10.1186/s12935-024-03358-6

**Published:** 2024-05-11

**Authors:** HaoYuan Wu, ZhiHao Yang, ChenXi Chang, ZhiWei Wang, DeRan Zhang, QingGuo Guo, Bing Zhao

**Affiliations:** Department of Neurosurgery, The Second Affiliated Hospital of Anhui Medical University, Anhui Medical University, 678 Fu Rong Road, Hefei, Anhui Province 230601 China

**Keywords:** Glioma, Disulfide death, importin-4, Migration, Invasion, Proliferation

## Abstract

**Background:**

“Disulfide death,” a form of cellular demise, is triggered by the abnormal accumulation of intracellular disulfides under conditions of glucose deprivation. However, its role in the prognosis of glioma remains undetermined. Therefore, the main objective of this study is to establish prognostic signature based on disulfide death-related genes (DDRGs) and to provide new solutions in choosing the effective treatment of glioma.

**Methods:**

The RNA transcriptome, clinical information, and mutation data of glioma samples were sourced from The Cancer Genome Atlas (TCGA) and the Chinese Glioma Genome Atlas (CGGA), while normal samples were obtained from the Genotype-Tissue Expression (GTEx). DDRGs were compiled from previous studies and selected through differential analysis and univariate Cox regression analysis. The molecular subtypes were determined through consensus clustering analysis. Further, LASSO analysis was employed to select characteristic genes, and subsequently, a risk model comprising seven DDRGs was constructed based on multivariable Cox analysis. Kaplan-Meier survival curves were employed to assess survival differences between high and low-risk groups. Additionally, functional analyses (GO, KEGG, GSEA) were conducted to explore the potential biological functions and signaling pathways of genes associated with the model. The study also explored immune checkpoint (ICP) genes, immune cell infiltration levels, and immune stromal scores. Finally, the effect of Importin-4(IPO4) on glioma has been further confirmed through RT-qPCR, Western blot, and cell functional experiments.

**Results:**

7 genes associated with disulfide death were obtained and two subgroups of patients with different prognosis and clinical characteristics were identified. Risk signature was subsequently developed and proved to serve as an prognostic predictor. Notably, the high-risk group exhibited an immunosuppressive microenvironment characterized by a high concentration of M2 macrophages and regulatory T cells (Tregs). In contrast, the low-risk group showed lower half-maximal inhibitory concentration (IC50) values. Therefore, patients in the high-risk group may benefit more from immunotherapy, while patients in the low-risk group may benefit more from chemotherapy. In addition, in vitro experiments have shown that inhibition of the expression of IPO4 leads to a significant reduction in the proliferation, migration, and invasion of glioma cells.

**Conclusion:**

This study identified two glioma subtypes and constructed a prognostic signature based on DDRGs. The signature has the potential to optimize the selection of patients for immune- and chemotherapy and provided a potential therapeutic target for glioma.

**Supplementary Information:**

The online version contains supplementary material available at 10.1186/s12935-024-03358-6.

## Introduction

Glioma, the most common malignant brain tumor, has high cellularity, mitotic activity, vascular growth, and necrosis [1]. Due to tumor invasiveness and chemotherapy and radiation resistance, glioma patients have significant recurrence and disability rates [2]. Tumor excision, adjuvant chemotherapy, and radiation are conventional glioma treatments [3]. Despite these treatments, glioma remains a complex clinical problem. Molecular biology has increased our understanding of glioma etiology and revealed relevant genetic abnormalities clinically [4]. Identifying more molecular markers is essential to more accurately assess glioma prognosis and explore more effective treatments.

Cell death is a physiological mechanism by which the organism maintains its normal functions through necrosis and programmed cell death (autophagy and apoptosis) [5]. With the discovery of cell death types such as ferroptosis [6] and pyroptosis [7], the relationship between cell death and diseases has regained attention. Recently, numerous studies have identified cell death-related biomarkers through integrated bioinformatic analyses to predict the prognosis of tumors. For instance, a panel comprising 4 LncRNAs associated with ferroptosis has been established to assess the prognosis of colon cancer patients [8]. In patients with hepatocellular carcinoma, Peng et al. identified a gene signature related to copper-induced cell death that holds significant value in predicting overall survival (OS) [9]. Furthermore, for glioma patients, a gene signature associated with ubiquitination exhibits the potential to predict disease outcomes [10]. However, research in the field of disulfide-related aspects in gliomas remains inadequate and incomplete.

Recently, a novel mode of cell death has been termed disulfide death. This particular cell death pathway results from the substantial accumulation of intracellular disulfide molecules. More specifically, the accumulation of cysteine leads to disulfide stress, inflicting damage upon the cells [11, 12]. Overcoming disulfide stress entails the reduction of NADPH levels to sustain cell viability. The primary source of cytosolic NADPH is the pentose phosphate pathway, which is derived from glucose. In cancer cells featuring an aberrant cysteine transporter known as solute carrier family 7 member 11 (SLC7A11; also referred to as xCT), rapid cysteine uptake and conversion to cystine, in conjunction with glucose deprivation, deplete the NADPH reservoir. This results in the massive accumulation of intracellular disulfide molecules and precipitates rapid cell death, characterized as “disulfide death” [13]. Hence, a bioinformatic exploration of DDRGs may shed light on their prognostic significance and unveil potential therapeutic targets for glioma treatment.

In the current study, we first screened DDRGs from glioma RNA sequencing data sourced from the TCGA and GTEx databases. Then, these genes identified two glioma subgroups exhibiting distinct clinicopathological and prognostic characteristics in both TCGA and CGGA databases. Next, a risk profile of 7 DDRGs was constructed to predict the prognosis of glioma patients. Immune cell infiltration, immunotherapeutic approaches, and chemotherapeutic interventions related to our glioma signature were comprehensively examined. Subsequent functional experiments revealed that knocking down IPO4 reduced the proliferation, migration, and invasion of glioma cells, indicating IPO4 as a potential innovative target for glioma therapeutic interventions.

## Materials and methods

### Data resources

**Fig. 1:**
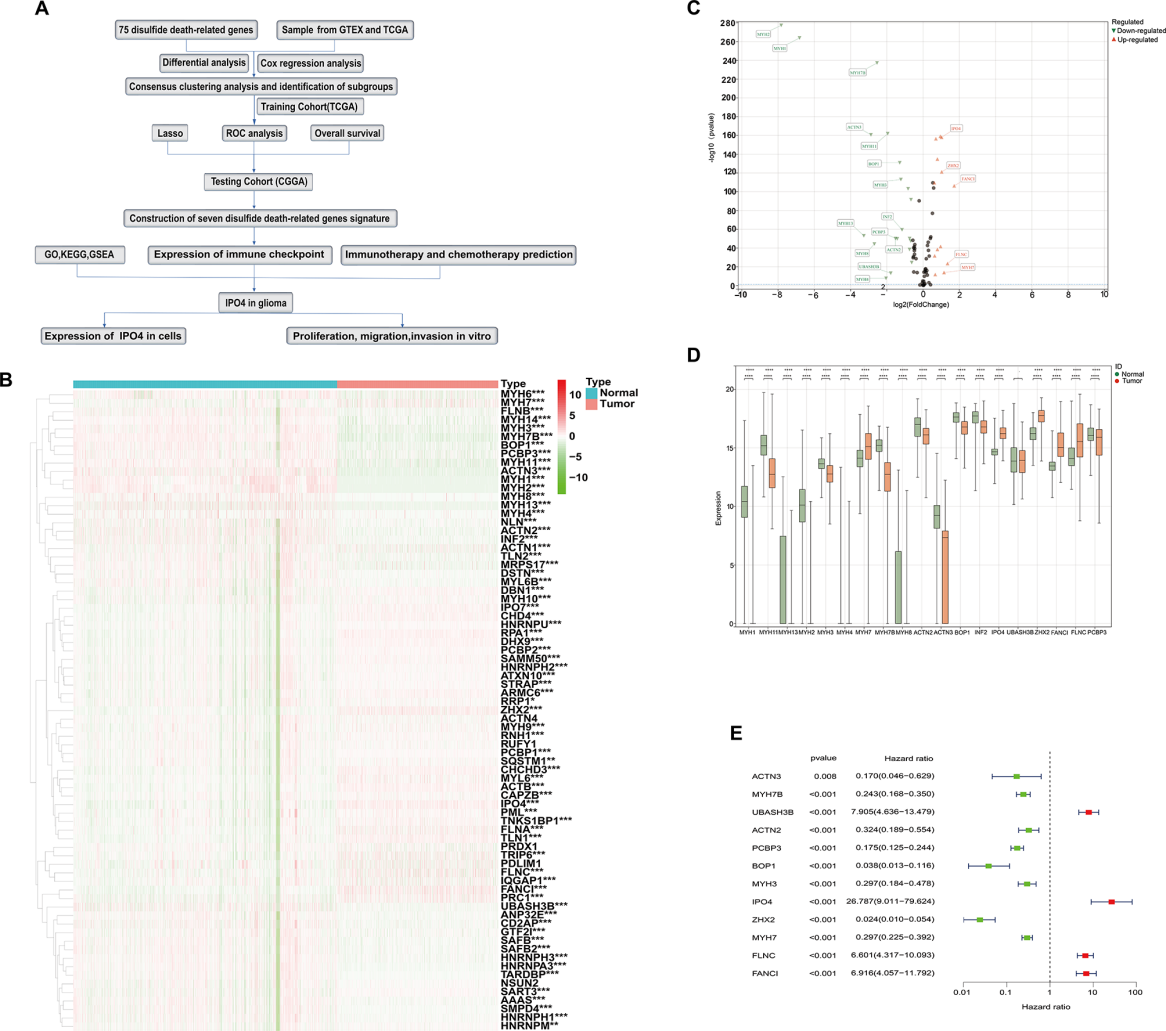
Workflow for the Acquisition, Identification, and Screening of DDRGs. **A** Workflow of the study. **B** Heatmap of 75 differentially expressed DDRGs. **C** Volcano plot with log2(FoldChange) absolute values greater than 1. **D** Differences in 19 DDRGs between glioma and normal samples. **E** Univariate Cox regression analysis

The workflow of this study is depicted in Fig. 1A, which involves the identification of DDRGs, molecular subtyping, and prognostic model construction in glioma. In addition, functional analysis, assessment of immunotherapy and chemotherapy effects based on the prognostic model, and subsequent validation of IPO4 through molecular and cellular experiments were conducted. As previously reported, we uncovered 75 genes related to disulfide mortality mediated by the actin cytoskeleton’s vulnerability to disulfide stress [13]. The CGGA and TCGA provided the datasets that we utilized in this study. The CGGA was accessed at http://www.cgga.org.cn/, while the TCGA can be accessed at https://portal.gdc.cancer.gov/. Additionally, some of the data was obtained from the GTEx project’s repository of standard tissue data. Following the use of data filtering techniques, a selection of datasets was made for further analysis. These datasets include TCGA-GBM, TCGA-LGG, and CGGA-693, as referenced in previous studies [14, 15]. The training group included the TCGA-LGG and TCGA-GBM datasets, whereas the validation set consisted of the CGGA-693 sample. After removing incomplete information, 670 glioma patients’ RNA-seq data and clinical information were obtained from TCGA, and 656 cases of glioma patients’ RNA-seq data and clinical information were obtained from CGGA. The Transcripts Per Kilobase Million (TPM) metric was derived by translating the original data obtained from TCGA or CGGA into Fragments Per Kilobase of Transcript Per Million Fragments Mapped (FPKM). The expression matrices were generated by applying quantile normalization and background correction to the datasets.

### Differential analysis

We used the “limma” package in RStudio (version 4.2.3) to do a differential analysis between TCGA glioma samples and normal brain samples. Differentially expressed genes (DEGs) were identified based on the criteria of p-value cutoff below 0.05 and absolute log2-fold change above 1. Subsequently, genes associated with the prognosis of glioma were selected through univariate Cox regression analysis, with a significance threshold set at *p* < 0.05.

### Consensus Clustering Analysis

The consensus clustering of glioma patients into distinct groups was performed using the R software “ConsensusClusterPlus.” The implementation of the cumulative distribution function (CDF) and consensus matrices was used in order to assess the optimal number of subgroups.

### Construction and validation of the risk signature

The “glmnet” software was utilized for LASSO regression analysis, considering the survival status, survival time, and expression levels of key genes in glioma patients. Subsequently, based on LASSO analysis, genes corresponding to lambda.min were selected for multivariable Cox analysis to construct the risk model. Finally, the model formula shown below was used to ascertain the risk score of each individual patient:

$$Risk\,score = \sum\nolimits_{i = 1}^n {coef\left( i \right) \times exp\left( i \right)}$$

In the above equation, the terms “coef($\varvec{i}$)” and “exp($\varvec{i}$)” represent the regression coefficients and gene expressions, respectively. Based on the median risk score, all cases were divided into groups that were considered low-risk and high-risk. Survival analysis was carried out via the use of KM curves. To evaluate the predictive accuracy of the 7 genes signature, time-related receiver operating characteristic (ROC) models were shown, and area under the curve (AUC) values were calculated by the “survivalROC” package in R. Each heatmap was generated with the R program “pheatmap.”

### Functional enrichment analysis

We performed a functional enrichment analysis of our risk signature using the R package “clusterProfiler” for Gene Ontology (GO) and Kyoto Encyclopedia of Genes and Genomes (KEGG) analyses. Using gene set enrichment analysis (GSEA) to determine functional enrichments by preparing GCT and CLS files, selecting the hallmark gene set, and running GSEA software (version 4.3.2). The results of functional enrichment in the high-risk group were obtained.

### Depiction of tumor microenvironment (TME) in glioma

We used the ESTIMATE method to evaluate the number of Stromal Scores, Immune Scores, and ESTIMATE Scores in order to further examine the tumor microenvironment (TME) in glioma [16]. Using the linear support vector regression (CIBERSORT) [17] deconvolution approach, the infiltration levels of tumor-infiltrating immune cells (TIICs) in each sample’s TME were determined. Significant differences in TIICs across various subgroups were found through comparisons.

### Analysis of tumor mutation burden (TMB), Immune checkpoints, and predictions for chemotherapeutic drugs

In the beginning, based on the work of Oslund et al. [18, 19], we gathered 38 ICP genes with therapeutic potential and confirmed their association with risk categories. Then, we utilized Perl to extract and process mutation data from glioma patients in the TCGA database. The Maftools package in R was employed to generate mutation waterfall plots for glioma patients from two distinct risk sets. We also compared the survival differences between high and low mutation burden groups. Additionally, chemotherapy drug predictions were conducted using oncoPredict to assess the IC50 values of chemotherapeutic drugs.

### Cell culture and treatment

The cell lines SNB19, SF126, LN18, T98G, U251, and U87 were obtained from the Chinese Academy of Sciences in Shanghai, China. These cell lines were followed by cultivation in DMEM and MEM media (Gibco, USA) supplemented with 10% fetal bovine serum (FBS). The glioma cells were cultured in a controlled environment with a humidified incubator set at temperature of 37°C and carbon dioxide concentration of 5%. General Biosystems created two siRNA molecules with the sequences 5′-CCTCGCAAGTTGTACGCAA-3’ and 5′-CCATGTTGGAAGAGGCTTT-3’ to specifically silence IPO4. After the cells in a 6-well plate achieved 60–70% confluence, 2 mL of DMEM and MEM media with 10% FBS were added to the growth medium to transfect the cells. Afterward, the JETPrime transfection reagent (Poly-plus-transfection®) was used to transfect cells to enhance siRNA import and accomplish IPO4 targeted silencing.

### RNA extraction and real‑time quantitative PCR

TRIzol reagent (Invitrogen; Thermo et al.) extracted total RNA from tissues and cells. Using a NanoDrop spectrophotometer (IMPLEN GmbH), the absorbance at 260/280 nm was used to measure the concentration and quality of RNA. After RNA was reverse transcribed into cDNA, real-time PCR (RT-qPCR) was carried out in an ABI 7500 real-time PCR system (Applied Biosystems) utilizing primers and TB Green mix (TaKaRa Biotechnology, China). Lastly, relative quantification was computed using GAPDH as the reference gene and the 2-DeltaDeltaCt technique. The PCR sequences employed in this research were as follows:

### GAPDH

forward:5’-AGCAAGAGCACAAGAGGAAG-3’;

reverse:5’-GGTTGAGCACAGGGTACTTT-3’.

### IPO4

forward:5’-TCCTTTGCCCCATTCTTTGC-3’;

reverse:5’-GCAAAGGACTTCTCTGCCAC-3’.

### Western blot

Using RIPA lysis buffer (Beyotime, China), the total protein from the treated glioma cells was extracted and then separated using SDS-PAGE. Following the transfer of proteins to a PVDF membrane (Millipore Corp, USA), a blocking step in the blocking solution (TBST with 5% skim milk) was conducted for three hours. After that, primary antibodies were incubated on the membrane for a whole night. Following three TBST membrane washes, TBST was mixed with a secondary antibody at a ratio of 1:10,000, and the mixture was incubated for one hour. After three further TBST washes, protein signals were finally detected using chemiluminescence detection kit (Thermo Scientific). The following antibodies were employed in the study: β-actin (Servicebio), IPO4 (Proteintech), PCNA (Proteintech), MMP10 (Proteintech), MMP9 (Proteintech) and Cyclin D1 (Proteintech).

### Cell counting kit-8 assay

Following cell treatment, 1000 U251, SF126, and U87 cells were planted in each well of 96-well plates. The cells were then continuously incubated for 0, 24, 48, and 72 h, respectively. The matching wells were filled with 10 µl of working solution (from Dojindo, Japan). The optical density (OD) values were determined at 450 nm after 2-hour incubation period using microplate reader.

### Cell migration and invasion assays

A total of 20,000 cells were seeded in a serum-free culture medium and subsequently introduced into Transwell chambers to conduct migration experiments. The Transwell chamber was positioned within 24-well plate that contained 600 µl of either DMEM or MEM with 30% FBS. Following a 24-hour incubation period, the cells on the upper surface of the chamber were carefully extracted. The cells were then fixed for 30 min with 4% paraformaldehyde and stained for 15 min with 0.5% crystal violet. 6 × 10^4 cells were introduced to chambers covered with matrix gel (Corning) for invasion experiments, and the cells were grown under the same conditions for 48 h. Similarly, cells were fixed for 30 min using 4% paraformaldehyde and stained for 15 min using 0.5% crystal violet. Eventually, an inverted microscope (Olympus, Japan) was used to take pictures.

### Statistical analysis

One-way analysis of variance (ANOVA) or t-tests were used to conduct comparisons of continuous variables across different groups. Each group’s OS was determined using Kaplan-Meier analysis. Statistical analysis was conducted using software tools such as RStudio and GraphPad Prism. Each experiment was repeated three or more times, and the results are shown as the mean value plus or minus the standard deviation. The symbols * indicate a p-value less than 0.05, ** indicate a p-value less than 0.01, and *** indicate a p-value less than 0.001. A significance level of *p* < 0.05 has been applied to determine the level of statistical importance.

### Results

#### Differential expression profile and screening of DDRGs

This figure (Fig. 1A) depicts the methodology used in our investigation. A notable disparity in the expression of 75 genes was observed between normal and glioma tissues (Fig. 1B). Furthermore, we conducted further refinement of the gene sets by selecting those with an absolute log2-fold change exceeding 1, as seen in Fig. 1C. Subsequently, an investigation was conducted to analyze the variations in these genes between glioma and healthy tissues, as seen in Fig. 1D. In this study, a univariate Cox regression analysis was employed to ascertain the correlation between twelve specific genes and prognosis, as depicted in Fig. 1E.

### Identification of glioma clusters using consensus clustering

**Fig. 2:**
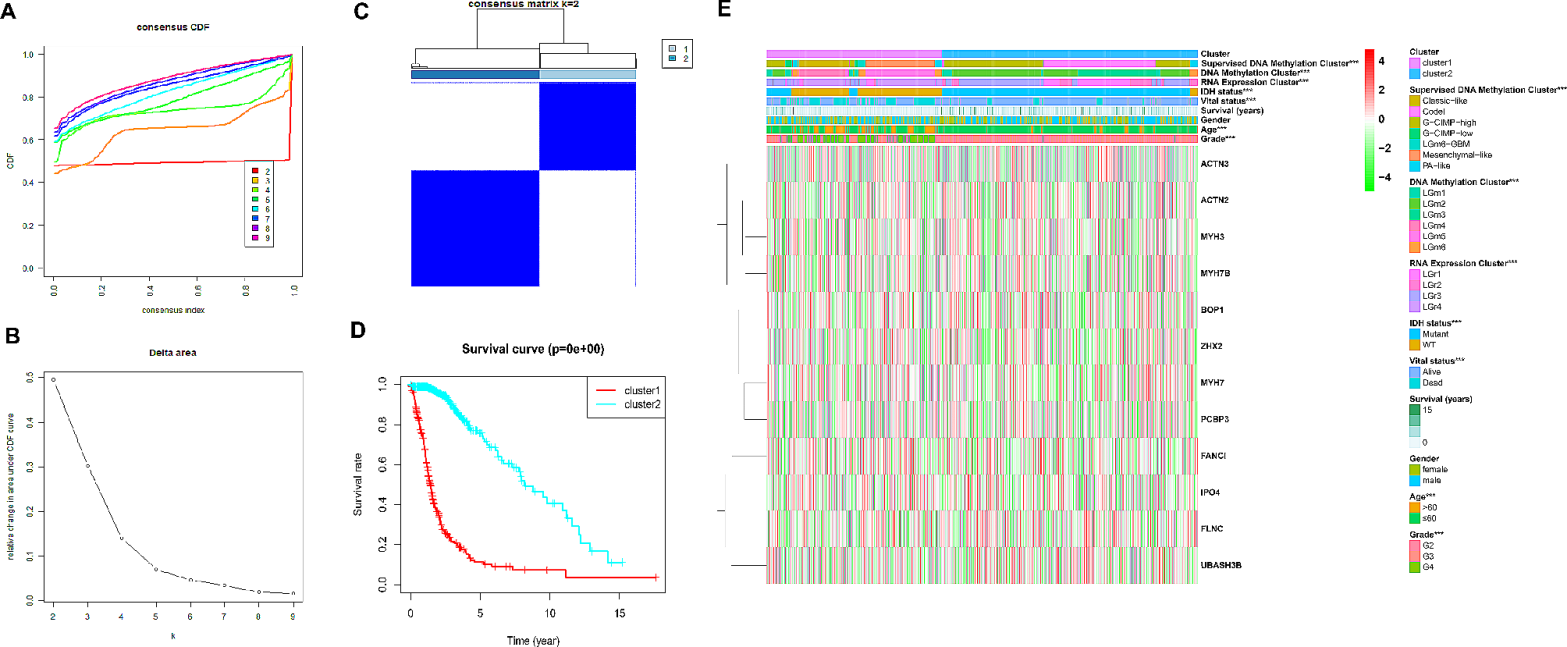
Clinical and Pathological Features of the Consensus Clusters of Glioma. **A** From k = 2 to 9, the CDF is used to find clusters of consistency. **B** CDF curve length and slope from 2 to 9. **C** All samples’ consistency score matrix at k = 2. **D** Kaplan-Meier graphs illustrate the disparity in OS between two subclusters within the training dataset. **E** Heatmap of DDRGs between the two clusters in the TCGA dataset

Cluster analysis was performed on 12 prognostic genes selected by univariate Cox analysis to identify different molecular subgroups. Figure 2A displays the CDF for different values of k, and the reliable choice is the value of k corresponding to the lowest CDF decline slope. Additionally, Fig. 2B reflects the relative change in the area under the CDF curve compared to k-1. When k = 2, the increase in the area under the curve is relatively small, suggesting that k = 2 might be a suitable choice for achieving clustering stability. The matrix heatmap also illustrates that the samples exhibit good clustering when k = 2 (Fig. 2C). Furthermore, it was noted that the individuals belonging to Cluster 1 had the most limited OS, as illustrated in Fig. 2D. We further investigated the molecular and clinical characteristics of two subgroups in conjunction with previously reported molecular subtyping studies [20]. According to Fig. 2E, there are notable differences between the two clusters in terms of age, glioma grade, survival status, IDH status, DNA Methylation Cluster, RNA Expression Cluster, and several other characteristics. Supplementary Figure S1 shows that glioma patients were divided into two stable subgroups based on the expression of 12 disulfide death-related genes in the CCGA database (Figure S1A-C). Cluster 1 subgroup has a short OS (Figure S1D). Combined with clinical data analysis, it was found that cluster 1 and cluster 2 subgroups were significantly correlated with glioma grade, age and survival status (Figure S1E).

### Risk signature for glioma constructed from 7 selected DDRGs

**Fig. 3:**
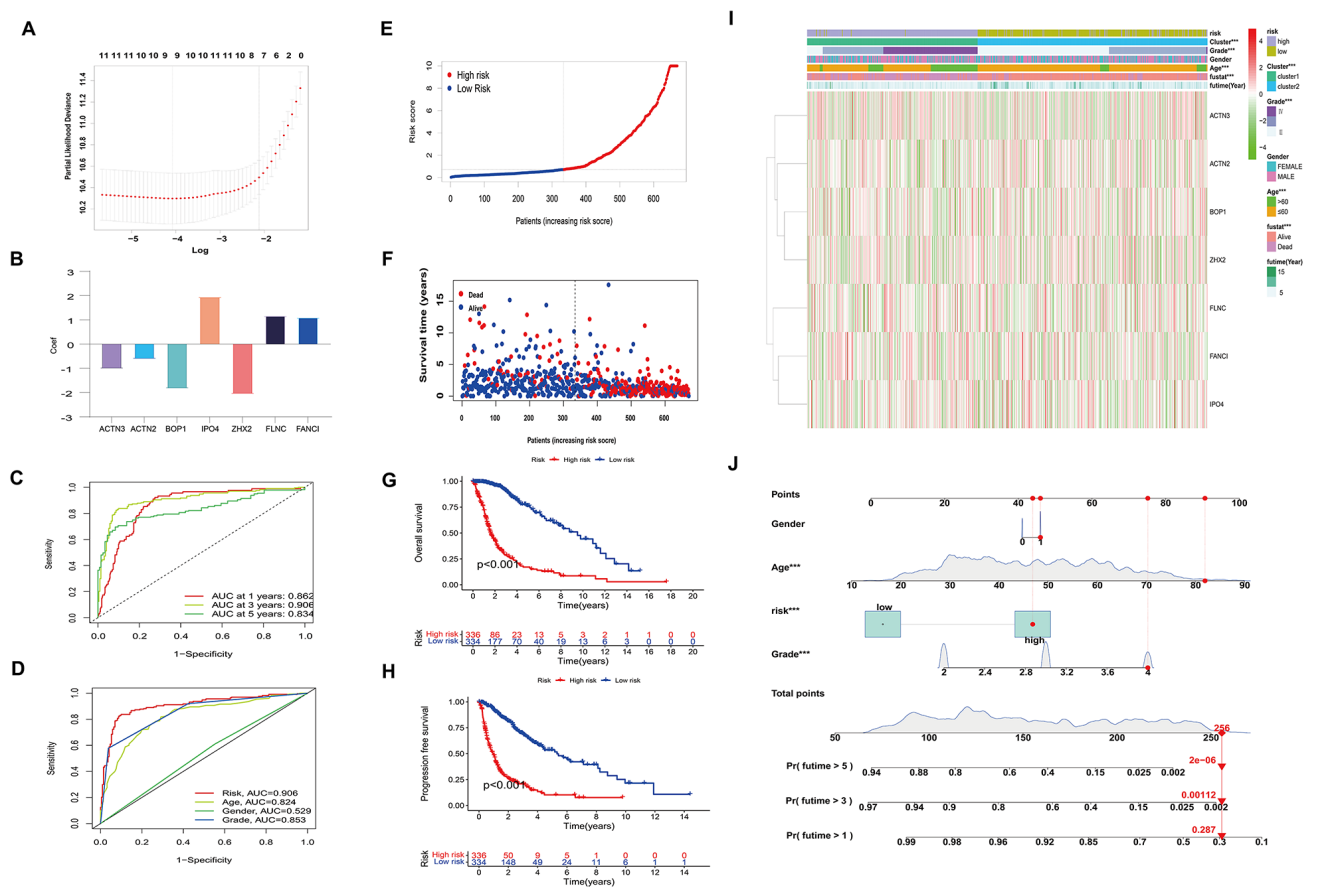
Identification of Prognostic DDRGs. **A** LASSO regression parameter selection cross-validation. **B** 7 DDRGs and their respective coefficients. **C, D** ROC curves measuring risk model predicting efficiency in the test sample. **E, F** Test set risk ratings and survival position. **G, H** Kaplan-Meier evaluation of survival of the 7 DDRGs between two test cohort risk groups. **I** Heatmap showing the expression levels of the 7 DDRGs and the distribution of clinical and pathological features in the high-risk and low-risk subgroups. **J** Nomogram predicting 1, 3, and 5-year survival rates of glioma patients based on risk and clinical factors

After conducting LASSO analysis, two specific λ values are indicated by the two dashed lines in Fig. 3A: one is lambda.min, and the other is lambda.1se. The λ values between these two points are considered appropriate. Lambda.min uses more genes and has higher accuracy. Here, we selected nine genes corresponding to lambda.min with higher accuracy for multivariable COX analysis (Fig. 3A), ultimately establishing a risk model consisting of seven genes (ACTN3, ACTN2, BOP1, IPO4, ZHX2, FLNC, and FANCI). Figure 3B displays the seven genes in the model and their corresponding coefficients. Subsequently, based on the expression levels of these genes, the risk scores for each glioma patient in the CGGA and TCGA databases were calculated. Using the median risk score as the threshold, glioma patients from both databases were categorized into high-risk and low-risk groups. In addition, it should be emphasized that the area of the validation set (as shown in supplementary Figure S2A-B) and the training set (as shown in Fig. 3C-D) under the ROC curve AUC is 0.7 above the threshold. The training cohort was divided into two risk groups based on the median risk score [Fig. 3E]. It is worth noting that high-risk patients have lower OS rates, as shown in Fig. 3G, and their survival time is also shorter, as shown in Fig. 3F. To confirm the accuracy of our model, we observed a relatively short progression-free survival in the high-risk group, as shown in Fig. 3H. Figure 3I shows significant differences between high - and low-risk categories associated with WHO grades, age, live status, and cluster groups. In addition, we predicted the probability of survival of patients in the next few years based on factors such as gender, age, risk level, and grade [Fig. 3J]. Similar results were obtained in the CGGA validation set, as shown in supplementary Figure S2C-G. The findings of this study indicate that a risk profile, derived from the 7 specific genes linked to disulfide mortality, might possibly function as a predictive biomarker for assessing the prognosis of individuals with glioma.

### Enrichment analysis of risk features

**Fig. 4:**
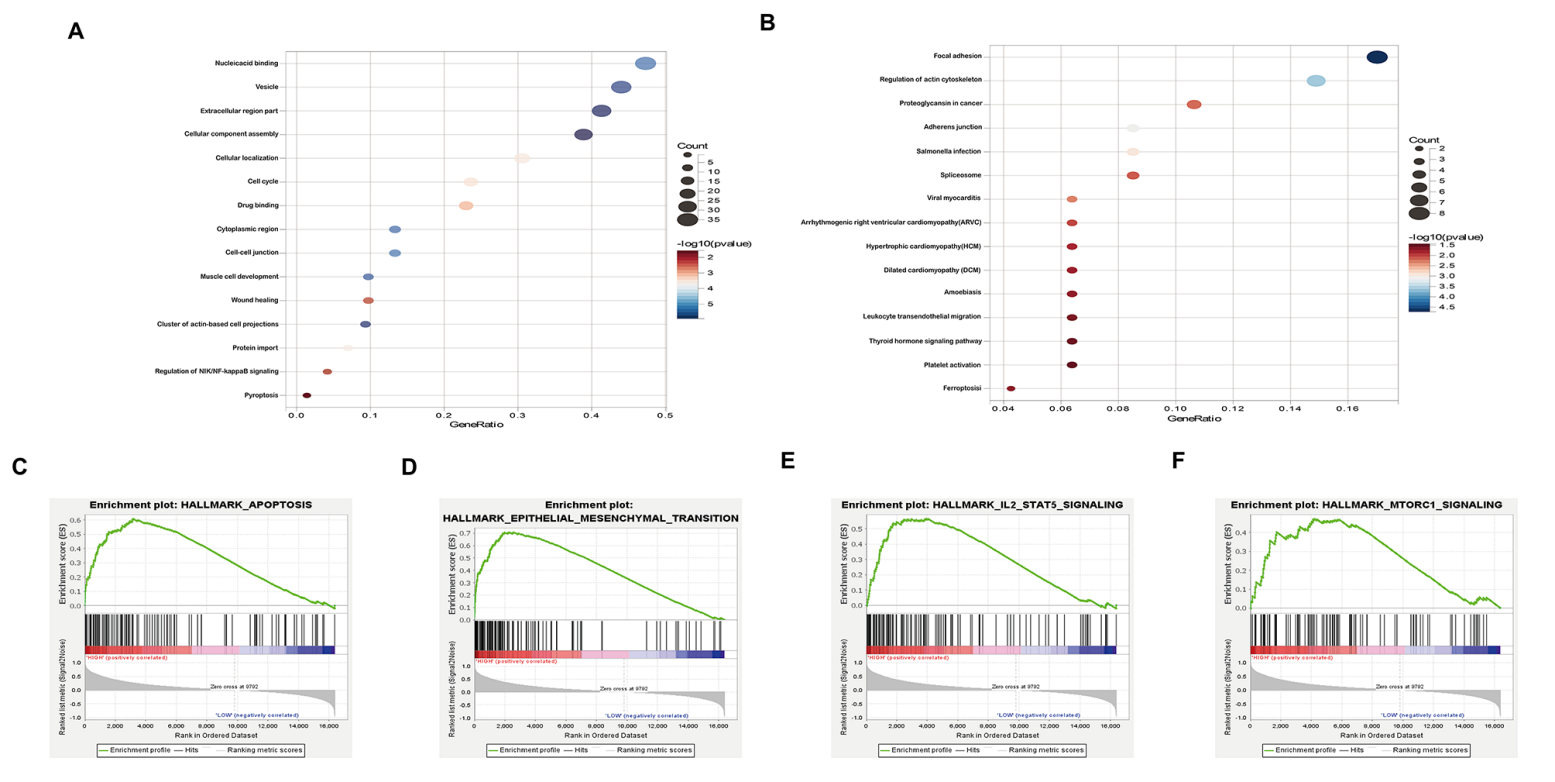
Functional enrichment analysis of risk-associated features. **A, B** GO enrichment (**A**) and KEGG pathway analysis (**B**). **C-F** The GSEA Analysis was conducted on the cohort from TCGA.

To examine potential variations in the functional characteristics of the 7 DDRGs, functional enrichment analysis was conducted on the groups categorized by risk. The GO enrichment analysis revealed a notable increase in cellular localization associated with the malignant progression of gliomas, as well as a correlation between cell cycle regulation and cell proliferation, control of the NF-kB signaling pathway, and cell-matrix adhesion (Fig. 4A). Furthermore, the KEGG pathway analysis revealed that leukocyte transendothelial migration, cytoskeletal modification, and focal adhesion were significantly enriched (Fig. 4B). The results of this study indicate a correlation between the malignant biological mechanisms of glioma and the associated risk factors. The high-risk group was demonstrated to be strongly linked with apoptosis, Epithelial-Mesenchymal Transition (EMT), MTORC1-signaling, and the IL-2_STAT5 signaling pathway, according to GSEA analysis (Fig. 4C-F).

#### Tumor microenvironment

**Fig. 5:**
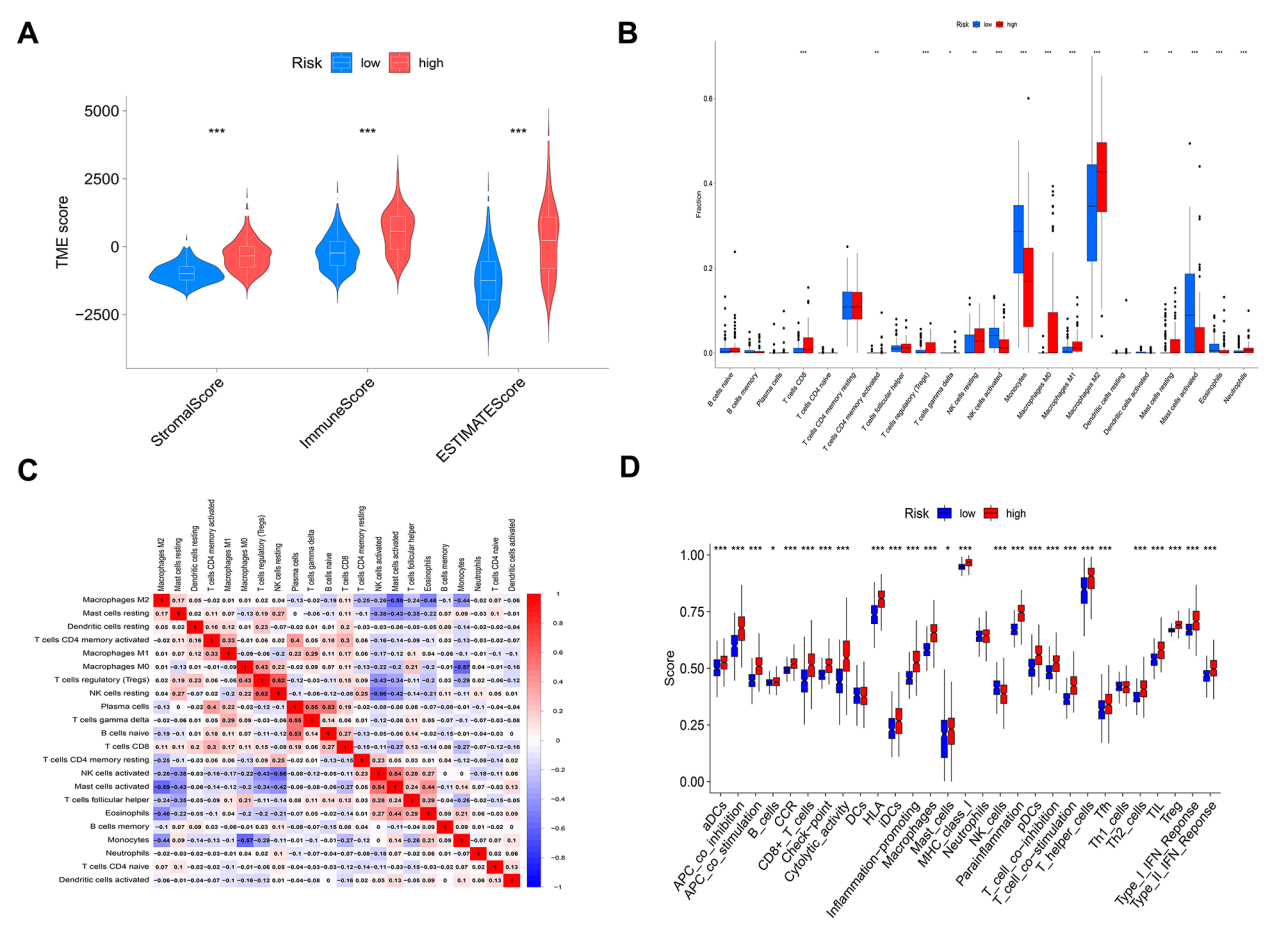
The evaluation of immune infiltration in relation to the risk model. **A** There exist notable disparities in immunological scores, stromal scores, and estimation scores between the two risk categories. **B** The presented visual representation is a bar chart that illustrates the percentages of tumor-infiltrating cells in two distinct risk categories. The low-risk group is denoted by the color blue, while the high-risk group is represented by the color red. **C** Co-expression study of cells that have invaded tumors within risk groups. **D** Bar chart comparing low-risk vs. high-risk immunological pathways

The assessment of markers for the TME was performed for each sample using the ESTIMATE technique. Subsequently, a comparison of TME characteristics was made between the two risk groups. Based on the results shown in Fig. 5A, it may be seen that the high-risk group had elevated scores in stromal, immunologic, and ESTIMATE evaluations. The quantification of immune cells in the two groups at risk was assessed by the ssGSEA algorithm. as seen in Fig. 5B. The findings of the study revealed that the high-risk group had an increase in the presence of M2 macrophages, CD8 + T cells, and Tregs. On the other hand, the group with low risk demonstrated an increase in the presence of activated natural killer (NK) cells, monocytes, and activated mast cells. The possible impact of immune cell interactions, as seen in Fig. 5C, on the effectiveness of immunotherapy has the capacity to influence the selection of treatment options. The correlation found between resting NK cells and Tregs is of particular significance. Furthermore, the heatmap depicted the distinct activation patterns of immune pathways in the low- and high-risk groups. This observation indicated significant disparities in immune pathways, such as the Type I and II IFN response, inflammation-promoting pathways, checkpoint inhibition, and T-cell co-stimulation pathways, between the two risk groups (Fig. 5D). The present study has shown the capacity to forecast the cellular immunological characteristics of gliomas by leveraging risk factors.

### Immune checkpoints and chemotherapy drug sensitivity

**Fig. 6:**
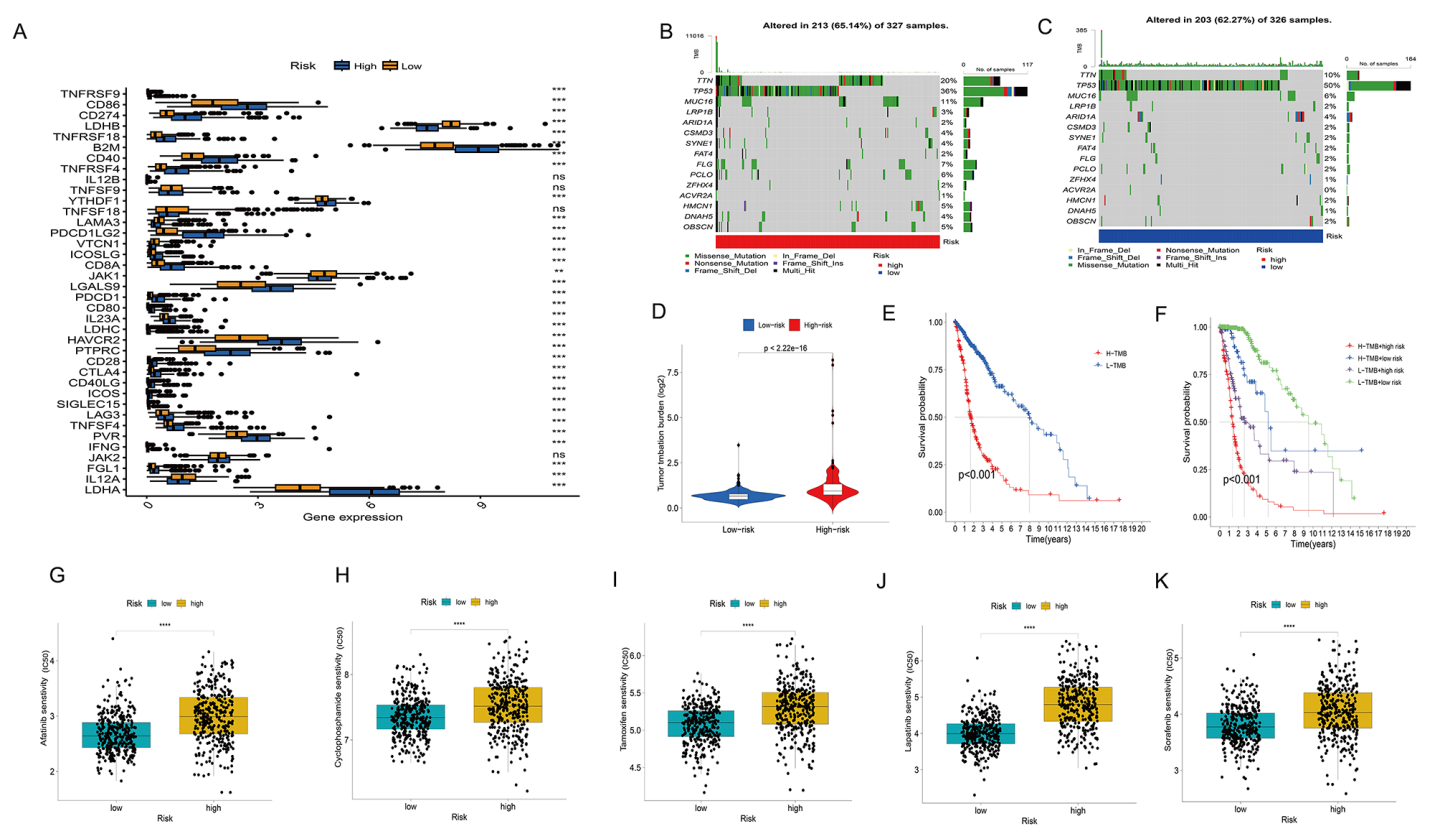
Differences in ICPs, Mutations, and Response to Chemotherapy between the Two Risk Groups. **A** A box plot was generated to visually represent the expression levels of 38 ICP molecules in the two risk groupings. **B, C** Waterfall plots are used to visually represent the relative mutation rates of the top 15 genes in both the high-risk (**B**) and low-risk (**C**) categories. **D** TMB scores in the low-risk and high-risk groups. **E** Kaplan-Meier analysis displaying OS rates in the low TMB and high TMB groups. **F** The Kaplan-Meier analysis reveals the OS rates within the low-risk and high-risk categories of both the low TMB and high TMB groups. **G-K** The markers of the dual sulfur death-related genes can serve as potential indicators for evaluating the sensitivity to Afatinib (**G**), Cyclophosphamide (**H**), Tamoxifen (**I**), Lapatinib (**J**), and Sorafenib (**K**)

In addition, we performed an assessment of the correlation between risk factors and ICP genes. The findings of this study revealed that there was a significant upregulation of PD-1(programmed cell death protein 1), CTLA4(Cytotoxic T lymphocyte associate protein-4), PD-L1(Programmed Cell Death Ligand 1), CD28, CD80, and CD86 in the high-risk group. Conversely, the low-risk group exhibited overexpression of LDHB, LAMA3, VTCN1, JAK1, and IL12A (Fig. 6A). The genes TTN, TP53, and MUC16 exhibited a notable frequency of mutations, above 10%, in both the high-risk and low-risk groups (Fig. 6B–C). In both cohorts, the TP53 gene exhibited a significantly elevated mutation frequency, with rates of 36% and 50% observed, respectively. The group at high risk had a higher tumor mutation load in the research on mutations (Fig. 6D). The group at greater risk demonstrated a decrease in long-term survival rates as the tumor mutation load rose, as seen in Fig. 6E-F. With regard to the present utilization of chemotherapeutic agents in the management of gliomas, our investigation sought to evaluate the efficacy of these pharmacological therapies in two separate risk categories. The results indicate that the low-risk population may benefit from commonly administered chemotherapy agents (Afatinib, cyclophosphamide, tamoxifen, Lapatinib, and Sorafenib; as shown in Fig. 6G-K), as demonstrated by predictive models for five distinct chemotherapy treatments.

#### Silencing of IPO4 inhibits proliferation and migration/invasion of glioma cells

**Fig. 7:**
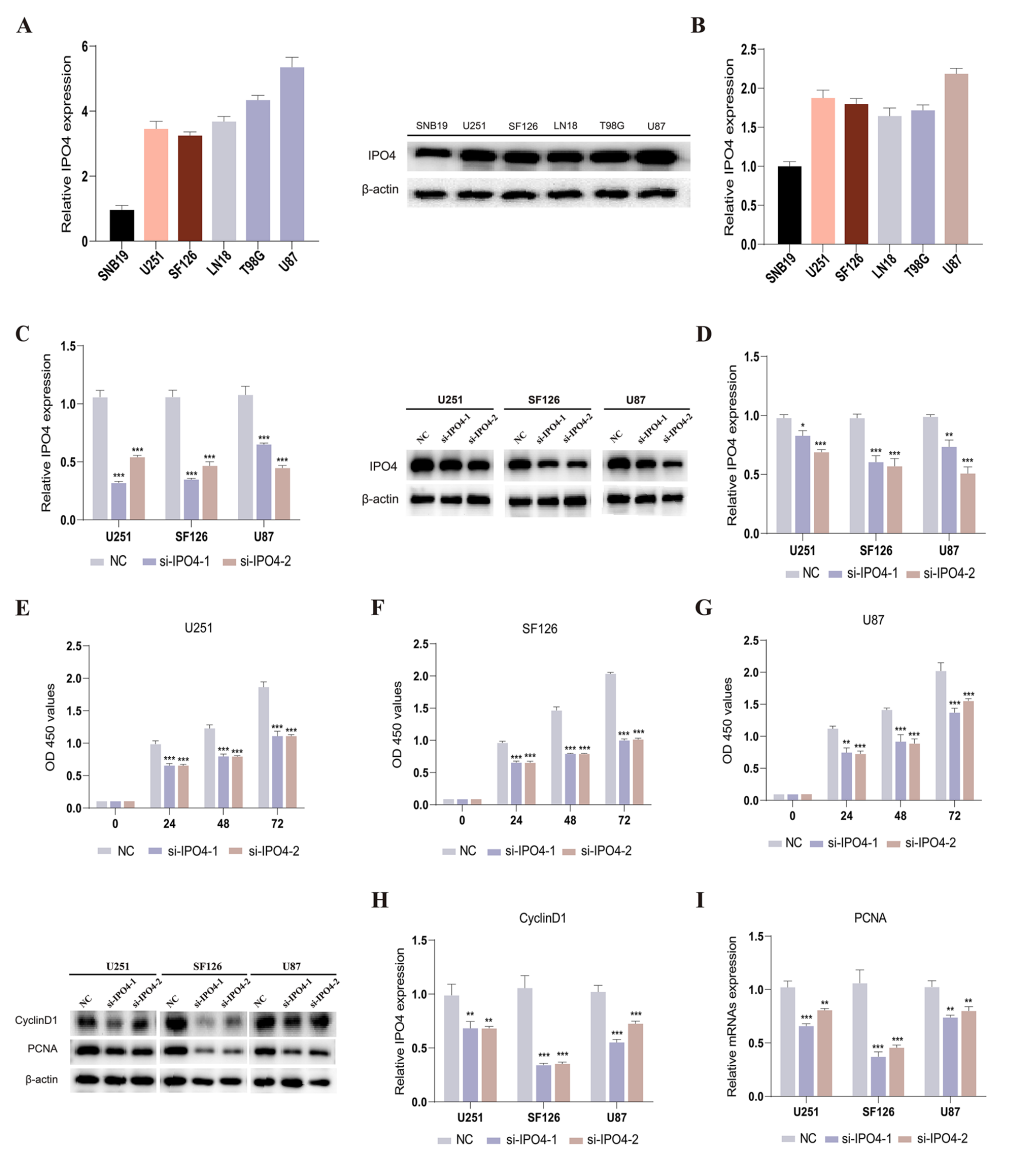
IPO4 Promotes In Vitro Proliferation of Glioma. **A, B** Relative expression of IPO4 in SNB19, U251, SF126, LN18, T98G, and U87 cells was determined by RT-qPCR (**A**) and Western blot (**B**). **C, D** Detection of IPO4 expression levels by RT-qPCR (**C**) and Western blot (**D**) when treated with IPO4 siRNA. **E-G** CCK-8 experiments indicated that silencing IPO4 inhibited the proliferation ability of U251 (**E**), SF126 cells (**F**), and U87 cells (**G**). **H, I** Western blot detected the differential expression levels of Cyclin D1 (**H**) and PCNA (**I**) proteins in the IPO4 silencing group and the control group. The biological tests were conducted independently and replicated a minimum of three times

**Fig. 8:**
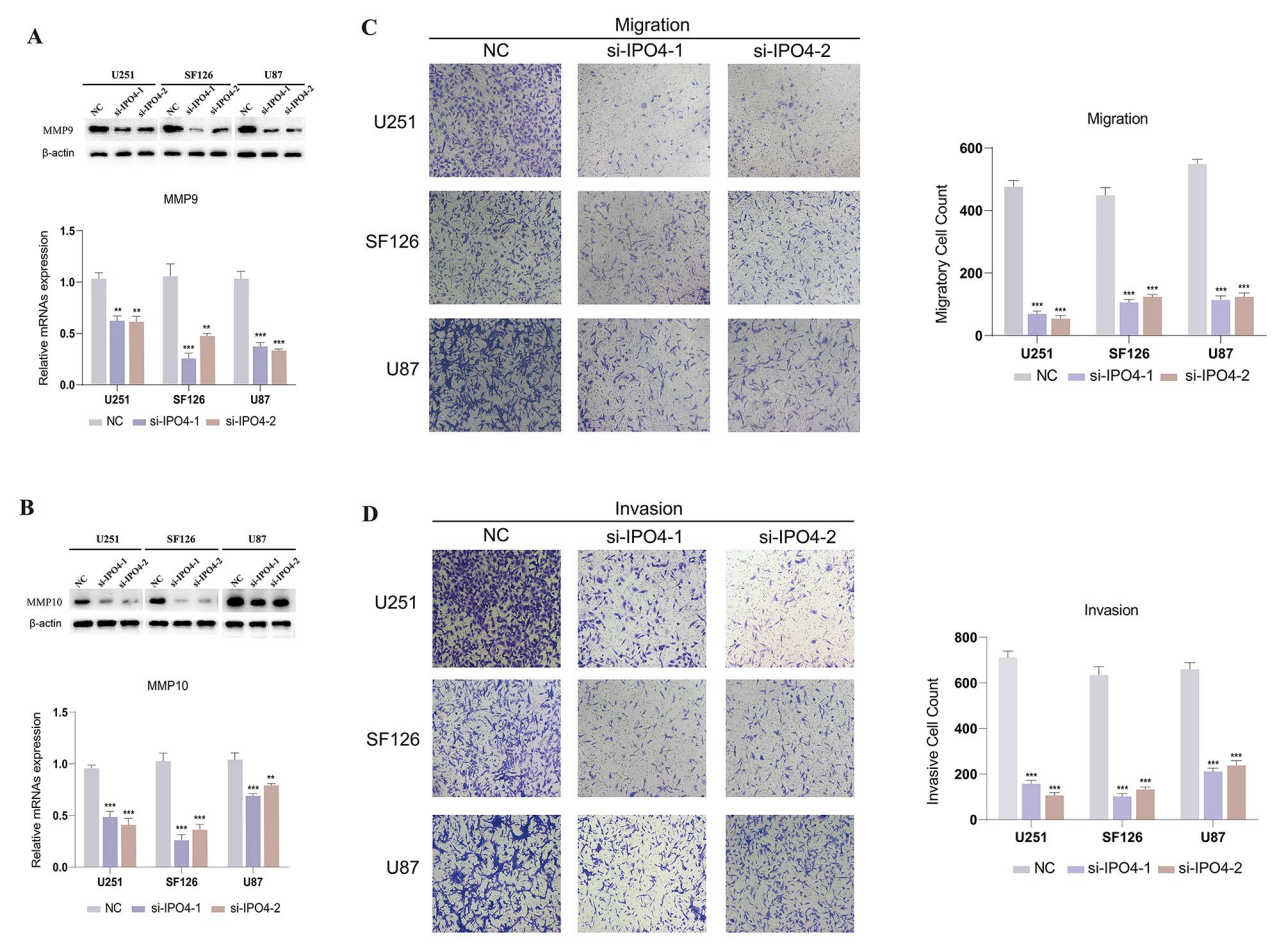
IPO4 promotes migration and invasion of brain glioma cells. **A, B** Western blot detected the differential expression levels of MMP9 (**A**) and MMP10 (**B**) proteins in the IPO4 silencing group and the control group. **C, D** The results of the Transwell studies demonstrated that the inhibition of IPO4 expression led to a significant reduction in the migratory (**C**) and invasive (**D**) capabilities of U251, SF126, and U87 glioma cells. The biological tests were conducted independently and replicated a minimum of three times

A research was undertaken to better explore the involvement of 7 DDRGs in gliomas. Based on our first investigation, it is evident that the gene IPO4 in glioma cells has yet to receive much attention in terms of functional testing. Consequently, a decision was made to conduct a functional analysis of gliomas using IPO4. Initially, we conducted a comprehensive analysis of the TCGA and GTEx databases to confirm the higher expression of IPO4 in glioma tissues compared to normal brain tissues. We performed an extensive examination of the TCGA and GTEx databases to validate the elevated expression of IPO4 in glioma tissues relative to normal brain tissues. Following that, the technique of real-time PCR was employed to evaluate the comparative degree of IPO4 activity in cell lines associated with glioma (as seen in Fig. 7A). Compared to other glioma cell lines, the expression of IPO4 was notably higher in the U251, SF126, and U87 cell lines (Fig. 7B). RT-qPCR was used to verify the knockdown effectiveness. The obtained findings indicated that IPO4 sequences 1 and 2 exhibited higher knockdown efficiency in U251, SF126, and U87 cell lines, as shown in Fig. 7C. Following this, small interfering RNA (siRNA) was employed to promote the downregulation of IPO4 in U251, SF126, and U87 cell lines. The confirmation of suppression effectiveness was achieved using Western blotting analysis, as depicted in Fig. 7D. The experimental findings from the CCK8 experiment provided evidence that the silencing of IPO4 resulted in a decrease in cell viability, as seen in Fig. 7E–G. Both Cyclin D1 and PCNA, which are often used as markers for cell proliferation, exhibit a consistent decrease in their expression levels, as seen in Fig. 7H-I. Finally, the results obtained from transwell tests demonstrated that the migratory and invasive capacities of glioma cells were significantly impaired when IPO4 expression was suppressed, as seen in Fig. 8C-D. Notable modifications are also seen in the cell migration markers MMP9 and MMP10, as shown in Fig. 8A-B. The results imply that IPO4 holds promise as a potential candidate for therapeutic interventions in glioma.

## Discussion

The most dangerous primary central nervous system tumor is glioma due to its high recurrence rate and fast malignant development [21]. The disease’s molecular heterogeneity makes glioma sufferers’ outlook grim. Understanding glioma genome alterations has improved predictive classification for customized therapy [22–24]. Despite advances in understanding the molecular processes of glioma, several molecular indications that might predict prognosis or guide treatment choices are still lacking [25]. The basic prognostic and therapeutic targets of glioma must be thoroughly studied.

Based on the expression profiles of 12 DDRGs, we identified two glioma subgroups with different prognoses and clinicopathological characteristics by applying consensus clustering analysis. Similarly, Zhang et al. identified two subgroups of lung adenocarcinoma using genes associated with the pulmonary basement membrane, which have been demonstrated to be effective prognostic factors [26]. Wang et al. also delineated two glioma subgroups based on cuproptosis-related genes [27]. It has been reported that patients with IDH wild-type present a poorer prognosis [28]. In our Cluster 1, a higher proportion of elderly, higher-grade, deceased, and IDH wild-type patients were observed, indicating a poorer prognosis. Additionally, previous studies have combined the mutational status of IDH to classify gliomas into different subtypes. Seven glioma subtypes were identified based on supervised DNA methylation cluster, six subtypes based on DNA methylation cluster, and four subtypes based on RNA expression cluster [20]. The G − CIMP − low, LGm5, and LGr4 groups individually exhibited poorer prognoses, being more enriched in the Cluster 1 subgroup in our classification, consistent with our results. Then, a prognostic risk signature with 7 DDRGs was established by performing the Lasso regression analysis and multivariable Cox analysis. Subsequently, the accuracy and predictive performance of this model were assessed and validated in two datasets, TCGA and CGGA, concerning the prognosis of these patients. The findings demonstrate the efficacy of this risk-based signature for prognostic predictions in glioma patients.

According to GO and KEGG analysis, DDRGs are associated with various aspects of cancer biology. These include crucial physiological functions including cell cycle, focal adhesion, and cytoskeletal regulation. Unrestrained reproduction and dispersion of aggressive glioma cells depend on cell cycle disruption [29]. Cytoskeletal mechanisms regulate glioma cell proliferation, epithelial-mesenchymal transition, migration, invasion, and immune evasion [30]. Focal adhesion has been extensively investigated in the context of cancer cell migration and metastasis. It links the cellular cytoskeleton to the extracellular matrix (ECM), providing the traction necessary for cell movement [31, 32]. GSEA showed that the high-risk group had higher levels of apoptosis and EMT. Furthermore, the IL-2-STAT5 signaling pathway was significantly enriched. Natural apoptosis eliminates damaged or faulty cells to prevent tumor growth. Anti-apoptotic proteins are upregulated by tumor cells to increase their viability [33]. Since many anticancer drugs induce apoptosis, tumor cell resistance may reduce therapeutic efficacy. Malignant epithelial cancers undergo EMT, which creates locally invasive and metastatic subtypes [34]. The IL-2-STAT5 signaling pathway is crucial to T cell proliferation, survival, differentiation, and immunological responses [35]. This technique is crucial to understanding immune system functions and its potential uses in cancer research and immunotherapy. Thus, the 7 DDRGs are likely to regulate several biological systems in glioma cells. These pathways include cell proliferation, EMT, immunological response, migration, and invasiveness.

Tumor growth is typically accompanied by the establishment of the TME, defined by alterations in nearby connective tissues and extracellular matrix, which ultimately create conducive environment for the survival of tumor cells [36]. Previous research has shown that non-tumor cells within the TME, including stromal and immune cells, significantly influence the progression of glioma [37, 38]. Stromal cells associated with glioma, including astrocytes and endothelial cells, play crucial roles in the mechanisms of tumor initiation, angiogenesis, and invasion [39]. To summarize the above points, it is evident that higher stromal score supports tumor cells, while higher immune score reflects active immune response to the tumor. Additionally, the ESTIMATE algorithm revealed that individuals in our high-risk group exhibited elevated stromal and immune scores. Studies on the TME have revealed that immune cells within tumors, particularly T cells and macrophages, as well as immune-regulatory molecules, have substantial impact on tumor progression and patient prognosis [40]. Analysis of immune infiltrating cells in our risk model indicates that the high-risk group exhibits higher levels of M2 macrophages and Tregs. Conversely, the low-risk group has more activated NK cells, monocytes, and mast cells. Furthermore, in our study, individuals in the high-risk group exhibit elevated levels of ICP markers such as PD-1, PD-L1, CTLA4, CD86, CD80, and CD28. Some studies suggest that Tregs can express PD-1, allowing them to maintain immune tolerance and suppress autoimmunity by interacting with PD-L1 [41]. Tregs can also suppress other immune cells through ICP pathways, such as CD28-CD80/CD86 [42]. Some tumor cells typically highly express PD-L1, promoting immune evasion [43]. Tumor-associated macrophages (TAMs) have the capability to promote various biological processes, including tumor cell invasion, migration, and vascular corruption. Among these, M2 macrophages can secrete inhibitory cytokines and chemical signals, including upregulation of PD-L1, to suppress T cell activity, thereby weakening the immune response. Additionally, M2 macrophages may also promote the generation and expansion of Tregs by secreting immune-inhibitory factors such as IL-10 and TGF-β, contributing to immune suppression. CTLA4 is primarily expressed in T cells and inhibits the activation of helper T cells (Th cells) [44]. NK cells are potent immune effectors, regulating their anti-tumor functions by balancing activating and inhibitory ligands on their cell surface. For example, the engagement of CD155 ligands has been shown to stimulate anti-tumor immune responses, especially those involving NK cells [45]. During the process of tumor development, both mast cells and mononuclear cells within the tumor microenvironment exhibit certain anti-tumor effects. Some studies suggest that in certain situations, mast cells can release immune cytokines and mediators like tumor necrosis factor (TNF) and interferons to activate immune cells and promote anti-tumor immune responses [46]. Monocytes can differentiate into macrophages that carry out anti-tumor functions in the tumor microenvironment [47]. In addition, the high-risk group exhibits the activation of both type I and type II IFN responses, which can promote inflammation [48]. Considering the differences in immune pathway expression between the low-risk and high-risk groups, it is reasonable to speculate that glioma patients with high-risk scores often have a more pronounced immune microenvironment infiltration.

In both high-risk and low-risk mutation models, TP53, TTN, and MUC16 genes exhibit the highest occurrence of variations, primarily comprising missense mutations. Notably, TP53 has the highest mutation frequency and is often associated with unfavorable prognoses [49]. Our results indicate that the high-risk group exhibits a higher mutation burden, and both the presence of a higher mutation burden and the high-risk status are associated with poorer OS. Furthermore, previous reports have suggested that a high mutation burden and high expression of ICPs are generally considered more beneficial for immunotherapy [50]. Chemotherapy is the most common treatment method for gliomas. We evaluated five commonly used chemotherapy drugs, namely, afatinib, cyclophosphamide, sorafenib, lapatinib, and tamoxifen [51–55], and the results indicate that high-risk patients exhibited higher IC50 for these drugs. Overall, our study indicates that high-risk patients may benefit more from immunotherapy, whereas low-risk patients may benefit from chemotherapy.

Our risk model includes 7 DDRGs, five of which have been previously studied in tumors(ACTN2, ACTN3, BOP1, ZHX2, and FANCI) [56–60]. However, the role of IPO4 in glioma is unknown. Within our prognostic model, IPO4 possesses the highest regression coefficient value, signifying its predominant contribution to the model. IPO4, a member of the importin β family, plays a crucial role in transporting cargo across the nuclear pore complex by interacting with nucleoporins. Following the outcomes of functional experiments, we verify that IPO4 promotes proliferation, EMT, migration, and invasion of glioma cells, suggesting that IPO4 may serve as a novel underlying therapeutic target for glioma.

Although the research have been reported to use DDRGs to construct prognostic models in glioma, our study is different [61]. First, a new molecular typing of glioma patients based on genes associated with disulfide death showed that glioma patients were divided into two subgroups with different prognostic and clinical characteristics. Second, previous studies developed a risk signature of DDRGs using LRPPRC, RPN1, and GSY1 genes. We developed a risk signature of genes associated with disulfide death using the ACTN3, ACTN2, BOP1, IPO4, ZHX2, FLNC, and FANCI genes. Finally, the gene IPO4 with the highest risk coefficient was selected from the risk model, and its role in glioma progression was further verified by real-time quantitative PCR (RT-qPCR), Western Blot analysis and cell function experiments in glioma cell lines. However, further research is required to explore the specific molecular mechanisms underlying the impact of DDRGs on gliomas. Furthermore, it is necessary to conduct a more extensive investigation into the role of these genes in gliomas via the use of animal models. In conclusion, our study has the potential to aid healthcare professionals in evaluating the prognosis of glioma patients and implementing personalized treatment strategies.

### Electronic supplementary material

Below is the link to the electronic supplementary material.

Supplementary Material 1: Fig. 1 Clinical and Pathological Features of the Consensus Clusters of Glioma. **A** From k = 2 to 9, the CDF is used to find clusters of consistency. **B** CDF curve length and slope from 2 to 9. **C** All samples’ consistency score matrix at k = 2. **D** Kaplan-Meier graphs illustrate the disparity in OS between two subclusters within the testing dataset. **E** Heatmap of DDRGs between the two clusters in the CCGA dataset.

Supplementary Material 2: Fig. 2 Identification of Prognostic DDRGs. **A, B** ROC curves measuring risk model predicting efficiency in the test sample. **C, D** Test set risk ratings and survival position. **E, F** Kaplan-Meier evaluation of survival of the 7 DDRGs between two test cohort risk groups. **G** Heatmap showing the expression levels of the 7 DDRGs and the distribution of clinical and pathological features in the high-risk and low-risk subgroups.

## Data Availability

The original contributions presented in the study are included in the article/supplementary material. Further inquiries can be directed to the corresponding authors.
